# Ophthalmology

**Published:** 1897-12

**Authors:** Alvin A. Hubbell

**Affiliations:** Buffalo, N. Y.


### Progress in Medical Science.

### OPHTHALMOLOGY.

CONDUCTED BY ALVIN A. HUBBELL, M. D., Buffalo, N. Y.

### KINDERGARTEN WORK AND EYESIGHT.

DR. CASEY A. WOOD, of Chicago, has (*New York Med. Jour., July 17, 1897,*) written an article especially for kindergartners, in which he has endeavored to show that the beginnings of damaged eyesight are laid in the early years of school life; the prevention of the evil concerns chiefly, therefore, the kindergartner and the teachers in the lower forms, for the trouble is often past remedy when the pupil enters the high grades. He submits the following suggestions:

1. Every kindergarten and every school should be provided with certain well-known and simple tests of vision, and no child of any age should receive instruction who has not good eyesight.

2. It should be a part of the teacher's duty in the public schools, and in some private institutions the teacher is the only guardian (in any sense) that the child possess, to note any defects of vision and have them corrected if possible.

3. No child with uncorrected or incorrigible defects should be allowed to use his eyes for any kind of close work before he is eight or nine years of age, lest worse things befall.

4. In the kindergartens the children should be taught only those things that demand the minimum employment of the accommodation for near work. Fröbel's "gifts" are sufficiently numerous and varied to enable both teacher and children to pass happy and profitable hours without damaging the precious inheritance of vision, and without inflicting defective eyes upon generations yet unborn.

5. Some kinds of instruction are in their nature unsuited for infant's eyes. I admire the work and teachings of such well-known authorities as Kate Douglas Wiggin and Nora Archibald Smith, and agree with them in many of their contentions. For example, I feel, with them, that it is questionable whether "children naturally incline to large movements in drawing," and that "they instinctively make pretty figures." I would certainly not allow them to engage in any kind of drawing, because the tendency always is, as with the grown-up folk, to indulge more and more in elaborate designs.

6. If one turns to the plates in the back of Josephine Jarvis's translation of Frederick Fröbel's *Spiel und die Spielgegenstände der Kinder*, or to the elaborate designs affixed to many similar text-books on kindergarten study, (Mrs. Rowland Hill's *Brush Work for the Kindergarten*, for example,) it will not be difficult to eliminate those occupations and studies that are palpably inimical to the eyesight of the child.

7. Speaking broadly, Fröbel's first four "gifts," and the uses to which they are ordinarily put in the kindergarten and the occupations to which they may give rise, are mostly devoid of harm so far as the eyes are concerned. They also suggest many *Mutter* and *Koselieder*, the use of which in kindergarten work is so much to be commended and leaves so little to be criticised that I sometimes ask myself whether with balls and blocks and the accompaniment of song and play kindergarten children might not be made to undergo a healthier development than that which the more complex and elaborate occupations subserve.

8. Above all do I deprecate certain occupations commonly recommended by and pictured in most of the latest kindergarten text-books. These are perforating cards, embossing, fine sewing, drawing in all its forms and phases, most kinds of paper interlacing, intricate paper cutting and folding, peas work, clay modeling, chain making (except when the links are very large), bead stringing, etc. These practices, however little indulged in, are almost certain to damage the eyesight of kindergarten children.

9. Among the less hurtful occupations—some of them harmless—are games not involving near work to any extent, slat interlacing (with wide slats of well-contrasted colors), sand work (especially if indulged in as German children use it out of doors), gardening, that Fröbel loved so well, building with large blocks, and the occasional use of simple apparatus, like Putnam's "busy work tiles."

---

AN EXPLANATION OF THE ACTION OF IRIDECTOMY IN GLAUCOMA.

IN THE *Progrès Médical* for May 29th there is a remarkable article, by Dr. Abadie, on the nature of glaucoma and on the way in which iridectomy proves beneficial in that disease. The author begins with the remark that permanent pathological changes must give rise to continuous and not to transitory symptoms ; hence a glaucoma, acute or subacute, that manifests itself by crises cannot be due to a permanent change in the sclero-corneal region, or to effacement of the irido-corneal angle, or to structural alteration of Fontana's space. Transitory disturbances, he adds, are necessarily due to the agency of the nervous system. The theory of the nervous origin of glaucoma has been maintained before, but always with a preponderating influence ascribed to the fifth pair. Recent discoveries, however, show that the trigeminal is purely a sensory nerve, charged exclusively with conveying peripheral impressions to the centers. The trophic influence over the eye attributed to it must, therefore, be ascribed to the fibers of the sympathetic which accompany it and take part in the formation of the ciliary nerves.

On the strength of these facts, Dr. Abadie undertakes to show that glaucoma depends on irritation of the vaso-dilator nerves of the eye, a temporary irritation in the form marked by crises and a lasting one in the chronic variety. The increased tension results from exaggerated repletion of the blood-vessels and perhaps, also, from a supersecretion of intraocular fluids dependent thereon.

The author refers to François Franck's statement that the vaso-dilator nerves of the eye have the same medullary origin and follow the same course as the dilator nerves of the pupil, and he adds that, therefore, it is not astonishing that the pupil should always be dilated in glaucoma, since irritation of the one set of nerves is accompanied by irritation of the other set. But the most striking proof that glaucoma is really occasioned by dilatation of the bloodvessels of the eye, he says, is furnished by the action of mydriatics and meiotics. Atropine, for example, always aggravates the glaucomatous crises, even if it does not occasion them, while eserine mitigates them.

In cases of acute or subacute glaucoma, iridectomy acts surely, and it is of service in all forms in which the functional troubles are intermittent. The following are M. Abadie's ideas of the way in which it acts: Under normal conditions the nervous currents which regulate the reciprocal relations of vascular dilation and constriction pass through the nervous plexus, situated in the median zone of the iris, in which terminate a certain number of ciliary filaments. When the vaso-dilator current is in the ascendant it reaches this plexus without interruption, and dilation of the vessels of the eye is the consequence. But, if the plexus has been cut, the excessive action of the dilator current ceases and order is restored. In iridectomy, then, it is not the mere excision of a bit of the iris that is effective, but the excision of a portion of the nervous plexus that it contains.

M. Abadie goes on to say that it is easy to demonstrate the truth of this explanation. If, he says, in a case of acute glaucoma, simply the pupillary sphincter or the ciliary periphery is subjected to the excision, the median zone containing the circular plexus being left intact, the operation is of no use. If, however, the excision is so done as to involve only the median zone, it is as effective as complete excision. It is of little consequence whether the piece of iris removed is wide or narrow, provided it includes the whole breadth of the iris; indeed, simple section without any excision would suffice.—Editorial *New York Medical Journal*, July 10, 1897.

